# Determinants of stunting among under-five children in Ethiopia: a multilevel mixed-effects analysis of 2016 Ethiopian demographic and health survey data

**DOI:** 10.1186/s12887-019-1545-0

**Published:** 2019-06-01

**Authors:** K. Fantay Gebru, W. Mekonnen Haileselassie, A. Haftom Temesgen, A. Oumer Seid, B. Afework Mulugeta

**Affiliations:** 1 Tigray National Regional State, Bureau of Science and Technology, Mekelle, Tigray Ethiopia; 2 School of Public Health, College of Health Sciences, Mekelle University, Mekelle, Ethiopia

**Keywords:** Stunting, Multilevel level, Individual factors, Community factors, Under-five children, Ethiopia

## Abstract

**Background:**

Childhood stunting is the most widely prevalent among under-five children in Ethiopia. Despite the individual-level factors of childhood stunting are well documented, community-level factors have not been given much attention in the country. This study aimed to identify individual- and community-level factors associated with stunting among under-five children in Ethiopia.

**Methods:**

Cross-sectional data from the 2016 Ethiopian Demographic and Health Survey was used. A total of 8855 under-five children and 640 community clusters were included in the current analysis. A multilevel logistic regression model was used at 5% level of significance to determine the individual- and community-level factors associated with childhood stunting.

**Results:**

The prevalence of stunting was found to be 38.39% in Ethiopian under-five children. The study showed that the percentage change in variance of the full model accounted for about 53.6% in odds of childhood stunting across the communities. At individual-level, ages of the child above 12 months, male gender, small size of the child at birth, children from poor households, low maternal education, and being multiple birth had significantly increased the odds of childhood stunting. At community-level, children from communities of Amhara, Tigray, and Benishangul more suffer from childhood stunting as compared to Addis Ababa’s community children. Similarly, children from Muslim, Orthodox and other traditional religion followers had higher log odds of stunting relative to children of the protestant community.

**Conclusions:**

This study showed individual- and community-level factors determined childhood stunting in Ethiopian children. Promotion of girl education, improving the economic status of households, improving maternal nutrition, improving age-specific child feeding practices, nutritional care of low birth weight babies, promotion of context-specific child feeding practices and narrowing rural-urban disparities are recommended.

## Background

A child with a height-for-age Z score (HAZ) less than minus two standard deviations below the median of a reference height-for-age standard is referred as stunted [1]. It reflects a process of failure to achieve the linear growth potential as a result of prolonged or repeated episodes of under-nutrition starting before birth [1]. It is further indicated as the irreversible outcome of inadequate nutrition and a major cause for morbidity during the first 1000 days of a child’s life [2]; which is considered as a better overall predictor of under-nutrition in children. Stunting affects large numbers of children globally and has severe short-and long-term health consequences including poor cognition and educational performance, low adult wages, lost productivity and increased risk of nutrition-related chronic diseases when accompanied by excessive weight gain later in childhood [3]. It is also a vicious circle; because women who were themselves stunted in childhood tend to have stunted offspring, creating an intergenerational cycle of poverty and reduced human capital that is difficult to break [4].

In Ethiopia, stunting is one the foremost necessary health and welfare issues among under-five children [5]. No matter the economic process and therefore the substantial decline of impoverishment within the past decades within the country, childhood stunting remains at a high level and continues to be a serious public health problem within the country [6]. The prevalence of stunting in 2000, 2005, 2011 and 2016 in under-five Ethiopian children was reported to be 58, 51, 44, and 38.4%, respectively [7]. It was estimated that average schooling achievement for a person who was stunted as a child in Ethiopia is 1.07 years lower than for a person who was never undernourished. Under-nutrition is implicated in 28% of all child mortality in Ethiopia. Child mortality associated with under-nutrition has reduced Ethiopia’s workforce by 8% [8].

According to several studies; factors such as sex [9–11], maternal education [11, 12], father education [13], maternal occupation [14, 15], household income [11, 16], antenatal care service utilization [14, 16, 17] source of water [18], colostrum feeding [19] and methods of feeding [15, 19] contribute to stunting in Ethiopia. However, most of the studies so far focus on individual-level factors affecting stunting rather than community-level factors. Studies that focus only on individual fixed effects factors could ignore group membership and focus exclusively on inter-individual variations and on individual-level attributes. In this case, it has the drawback of disregarding the potential importance of group-level attributes in influencing individual-level outcomes. In addition, if outcomes for individuals within groups are correlated, the assumption of independence of observations is violated, resulting in incorrect standard errors and inefficient estimates [20]. However, multilevel study design allows the simultaneous examination of the effects of group-level and individual-level predictors [21]. Thus, this study was designed to identify both the individual- and community-level factors that contribute to stunting in Ethiopia.

## Methods

### Data sources

A cross-sectional data were obtained from 2016 Ethiopian Demographic and Health Survey (EDHS). The EDHS data had been collected by the Ethiopian Central Statistical Agency (ECSA) from January 18, 2016, to June 27, 2016 [22].

### Sampling procedures

A proportional sample of 15,683 households from 645 clusters was enclosed within the data assortment. The samples were stratified, clustered and designated in two stages. Within the 1st stage, 645 clusters (202 urban and 443 rural) were designated from the list of enumeration areas supported the 2007 Population and Housing Census sample frame; and within the second stage, 28 households per cluster were designated. Overall, 18,008 households were selected; of that 17,067 were occupied. Of the occupied households, 16,650 were with success interviewed, yielding a response rate of 98%. within the interviewed households, 16,583 eligible ladies were known for individual interviews; and interviews were completed with the eligible ladies, yielding a response rate of 95%. For this study, a total of 10, 641 children less than 59 months were identified in the households of selected clusters. Among whom, the complete height-for-age record was collected from 8855 children and 640 clusters. The remaining 1786 children and 5 clusters had missing values on height-for-age records. Thus, the analysis of this study was based on the 8855 under-five children.

### Outcome variable

The outcome variable was stunting, standing among children below 5 years as outlined by height-for-age < − 2 z scores relative to World Health Organization standards [23]. Stunting of the i^th^ child was measured as a dichotomous variable:

$$ Yi=\left\{\begin{array}{c}0,\kern0.5em Normal\ if\ z- score\ge -2 SD\  from\ the\ \mathrm{median}\ \mathrm{of}\ \mathrm{the}\ \mathrm{WHO}\ \mathrm{standards}\\ {}1,\kern0.5em Stunted\ if\ z- score<-2 SD\  from\ the\ \mathrm{median}\ \mathrm{of}\ \mathrm{the}\ \mathrm{WHO}\ \mathrm{standards}\end{array}\right. $$

Y_i_ = represent the stunting status of the *i*^*th*^ child.

### Independent variables

The independent variables for this study were elite supported previous studies conducted on the factors influencing childhood stunting at the worldwide and also the country level that were reviewed from the literature as determinants of stunting [14, 24, 25]. The variables were classified into two levels; individual- and community-level factors.

### Individual-level factors

Age of child, sex of the child, mother’s body mass index, age of the mother, maternal education, father’s education, wealth index, mother’s marital status, mother’s perceived size of the child at birth, child had diarrhea, child had fever in the last weeks, place of delivery, number of under-five children in the household, antenatal care visits, mother’s age at 1st birth, mother’s occupation, father’s occupation, birth type, preceding birth interval and mass-media exposure. Since the study was conducted among under-five children and the majority of the data indicated that child breastfeeding was until the children reach 2 years old, leading to the presence of high missing value, child breastfeeding status was not included. Statistical analysis is likely to be biased when more than 10% of data are missing [26].

### Community-level factors

Religion, region, place of residence, the source of drinking water, sanitation facilities, type of toilet, community women institutional delivery, community women education, and community women poverty were identified as community-level variables.

### Data analysis procedures

For the hierarchical structure of the EDHS data, multilevel multivariable logistic regression analysis was used. Once weight every variable, descriptive statistics were reported with frequency and proportion. The degree of crude association for individual and community characteristics was checked by employing a χ2 test. The fixed effects of individual determinant factors and community distinction on the prevalence of stunting were measured using an adjusted odds ratio (AOR). Within the multilevel multivariable logistical regression analysis, four models were fitted for the result variable. The primary model (empty or null model) was fitted without explanatory variables. The second model (individual model), third model (community model) and fourth model (final model) variables were fitted for individual-level, community-level, and for each individual- and community-level variable respectively. The ultimate model was used to check for the independent effect of the individual- and community discourse variables on childhood stunting. The data were analyzed using the STATA statistical software system package version 14.0 (StataCorp., college Station, TX, USA). It was considered statistically significant if the *P*-values less than 0.05 with the 95% confidence intervals.

### The goodness of fit test

Akaike information criterion (AIC) and the Bayesian information criterion (BIC) were used as regression diagnostics to determine the goodness of fit of the model; since stepwise methods were used to compare models containing different combinations of predictors. According to Boco [27], the AIC is calculated as − 2 (log-likelihood of the fitted model) +2p, where *p* is the degree of freedom in the model; and BIC assesses the overall fit of a model and allows the comparison of both nested and non-nested models which is calculated as − 2 (log-likelihood of the fitted model) + ln (N)*P. After the values for each model of AIC and BIC were compared, the lowest one thought-about to be a better explanatory model [28].

Multicollinearity amongst the individual- and community-level variables was checked using the Variance Inflation Factor (VIF). The mean value of VIF < 10 was cut off point [29].

## Results

### Bivariate analysis of the effects of multilevel factors on childhood stunting

The prevalence of childhood stunting was 38.39%; of that 34.81% was for females and 37.93% for males (Table 1). Throughout the bivariate logistic regression analysis, individual characteristics such as live births between births, preceding birth interval, number of under-five children in the household, fever and diarrhea conditions of the child, marital status of women, and maternal occupation were not significantly associated with childhood stunting at *p* < 0.05 (Table 1). However, all the community characteristics were found to be significantly associated with childhood stunting (*p* < =0.05) except for the comminity women education level and type of toilet (Table 1). Children of women living in communities with comparatively lower level of institutional delivery had relatively higher (39.57%) proportion of childhood stunting than those with higher institutional delivery (31.05%).

**Table 1:** Bivariate analysis of the effects of individual- and community-level factors on childhood stunting less than 5 years in Ethiopia, EDHS 2016

Individual- and community-level characteristics	Weighted sample and stunted (%)	*p*-value
Current child age		
0–5 moths	1079 (16.21)	*P* < 0.001
6–11 moths	999 (17.16)	
12–23 moths	1907 (40.89)	
24–59 moths	5575 (45.74)	
Child sex		
Male	4947 (37.93)	*P* = 0.002
Female	4344 (34.81)	
Live births between births		
No	7770 (38.98)	*P* = 0.46
Yes	73 (29.38)	
Type of birth		
Single	9364 (37.98)	*P* < 0.001
Multiple	226 (55.45)	
Preceding birth interval		
< =12 months	224 (52.53)	*P* = 0.60
> 12 months	7607 (38.52)	
Number of under-five children in the household		
<=2 children	9513 (38.36)	*P* = 0.093
> = 3 children	75 (42.74)	
Size of child at birth		
Large	2998 (34.83)	*P* < 0.001
Medium	1507 (37.28)	
Small	2487 (44.66)	
Child has diarrhea in the last week		
No	8418 (38.11)	*P* = 0.06
Yes	9150 (40.79)	
Child had fever recently		
No	8168 (37.91)	*P* = 0.141
Yes	1411 (41.46)	
Age of Women		
15–24 years	2198 (38.46)	*P* = 0.004
25–34 years	5137 (37.38)	
35–49 years	2335 (40.56)	
Women education level		
No education	6294 (41.63)	*P* < 0.001
Primary education	2615 (35.34)	
Secondary and above	679 (19.09)	
Marital status of women		
Single	50 (37.50)	*P* = 0.979
Married	9108 (38.23)	
Separated	424 (40.62)	
Maternal occupation		
No	6993 (38.25)	*P* = 0.540
Yes	2595 (38.77)	
Mothers body mass index		
Over weight	589 (24.44)	*P* < 0.001
Normal weight	7038 (38.84)	
Under weight	1865 (41.09)	
Number of antenatal care visits		
No visits	2465 (39.79)	*P* < 0.001
1–3 visits	2132 (37.82)	
4–20 visits	2160 (30.68)	
Place of delivery		
Institution	2688 (31.11)	*P* < 0.001
Home	6900 (41.23)	
Age of mother at first birth		
11–18 years	4807 (39.44)	*P* < 0.001
19–25 years	4394 (37.72)	
26–32 years	360 (33.40)	
33–40 years	27 (28.70)	
Father education level		
No education	4299 (42.62)	*P* < 0.001
Primary education	3650 (36.52)	
Secondary and above	1092 (25.73)	
Father’s occupation		
No	8927 (38.46)	*P* = 0.007
Yes	660 (37.46)	
Wealth index		
Poor	4291 (43.39)	*P* < 0.001
Middle	1974 (35.95)	
Rich	3322 (31.86)	
Mass media exposure		
No	1156 (26.86)	*P* < 0.001
Yes	8045 (37.79)	
Birth order of the last birth		
First	1753 (36.11)	*P* < 0.001
2nd and 3rd	2483 (37.04)	
4th and 5th	2291 (39.41)	
6th and above	2588 (40.57)	
Religion		
Protestant	2061 (36.93)	*P* = 0.003
Muslim	3909 (36.95)	
Orthodox	3327 (40.16)	
Others	290 (47.95)	
Place of residence		
Urban	1048 (26.15)	*P* < 0.001
Rural	8540 (39.89)	
Region		
Addis Ababa	211 (14.68)	*P* < 0.001
Afar	91 (40.66)	
Amhara	1878 (47.17)	
Oromia	4203 (36.25)	
Somali	397 (26.98)	
Benishangul	101 (42.83)	
SNNPR	1978 (39.12)	
Gambella	22 (23.33)	
Harari	18 (31.85)	
Tigray	549 (38.85)	
Dire Dawa	36 (41.22)	
Source of drinking water		
Unimproved	4134 (40.66)	*P* = 0.002
Improved	5310 (36.86)	
Source of type of toilet		
Unimproved	5078 (36.65)	*P* = 0.054
Improved	4397 (40.48)	
Community women poverty		
Low	5511 (36.88)	*P* < 0.001
High	4076 (40.44)	
Community women institutional delivery		
Low	5560 (39.57)	*P* < 0.001
High	3295 (31.05)	
Community women primary education		
Low	4789 (39.38)	*P* = 0.630
High	4799 (37.41)	

### Multivariable multilevel logistic regression analysis of individual-level factors

During multivariable multilevel analyses, factors such as age and sex of the child, maternal education, wealth index, birth type, size of the child at birth, and maternal body mass index were found to be independently associated with the odds of childhood stunting (Table 2). The log odds of stunting was higher among children in the age group of 12–23 months and 24–59 months (AOR = 5.04, 95%CI: 3.95–6.41) and (AOR = 10.00, 95%CI: 7.71–12.98) respectively as compared to the age group of 0–5 months age. Moreover, female children were less likely to be stunted (AOR = 0.85, 95%CI: 0.75–.94) as compared to males. Similarly, the odds of stunted children of single births were 47% (AOR = 0.53, 95%CI: 0.32–0.89) less likely than those multiple births. The large size children at birth were less likely than the odds of childhood stunting among children with small size (AOR = 1.68, 95%CI: 1.40–2.00) and medium size (AOR = 1.20, 95%CI: 1.02–1.40), after controlling for other individual- and community-level variables in the model (Table 2). Similarly, mothers with secondary and above education were 27% (AOR = 0.73, 95%CI: 0.57–0.95) less likely to have stunted children compared to those with no education. The odds of childhood stunting were also higher among children from underweight mothers compared to those from overweight mothers (AOR = 1.56, 95%CI: 1.17–2.08). Children from the rich households were 34% (AOR = 0.66, 95%CI: 0.54–0.79) less likely to be stunted compared to children from poor households.

**Table 2:** Multivariate multilevel logistic regression model of the effects of individual- and community-level factors on child stunting less than five years in Ethiopia, EDHS 2016

Individual- and community-level characteristics	Null model	Model 2	Model 3	Model 4
	Empty model	Individual-level variables	Community-level variables	Individual- and community-level variables
		AOR(95% CI)	AOR(95% CI)	AOR(95% CI)
Current child age				
0–5 moths (reference)		1		1
6–11 moths		1.29(0.97–1.71)		1.27(0.95–1.69)
12–23 moths		5.1***(3.97–6.38)		5.04***(3.95–6.41
24–59 moths		10.***(7.78–2.97)		10***(7.71–12.98)
Child sex				
Male (reference)		1		1
Female		0.85(0.75–0.96)		0.85*(0.75–0.94)
Type of birth				
Multiple (reference)		1		1
Single		0.53*(0.32–0.89)		0.50*(0.30–0.85)
Size of child at birth				
Large (reference)		1		1
Medium		1.19*(1.02–1.38)		1.20*(1..02–1.40)
Small		1.7***(1.44–2.02)		1.68***(1.40–2.00)
Age of Women				
15–24 years (reference)		1		1
25–34 years		0.94(.77–1.14)		0.96(0.78–1.18)
35–49 years		0.92(0.70–1.22)		0.93(0.71–1.24)
Women education level				
No education (reference)		1		1
Primary education		0.95(0.80–1.12)		0.95(0.80–1.13)
Secondary and above		0.69*(0.51–0.93)		0.73*(0.57–0.95)
Body mass index				
Over weight (reference)		1		1
Normal weight		1.51**(1.17–01.94)		1.34*(1.03–1.75)
Under weight		1.74***(1.32–2.3)		1.56**(1.17–2.08)
Number of antenatal care visits				
No visits (reference)		1		1
1–3 visits		1.02(0.87–1.20)		0.98(0.84–1.17)
4–20 visits		0.87(.73–1.04)		0.83(0.68–1.00)
Place of delivery				
Institution (reference)		1		1
Home		1.08(0.92–1.28)		1.04(0.87–1.24)
Age of mothers at first birth				
11–18 years (reference)		1		1
19–25 years		0.99(.87–1.14)		1.02(0.90–1.17)
26–32 years		1.07(0.76–1.52)		1.06(0.74–1.52)
33–40 years		1.02(0.39–2.65)		1.01(0.36–2.80)
Father education level				
No education (reference)		1		1
Primary education		0.83*(0.71–0.96)		0.86(0.73–1.00)
Secondary and above		0.72**(0.57 0.90)		0.85(0.67–1.08)
Wealth index				
Poor (reference)		1		1
Middle		0.89(0.75–1.1)		0.83(0.68–1.01)
Rich		0.72***(0.6–0.85)		0.66***(0.54–0.79)
Mass media exposure				
Yes (reference)		1		1
No		1.12(0.90–1.38)		0.99(0.79–1.25)
Birth order of the last birth				
First (reference)		1		1
2nd and 3rd		0.91(0.74–1.12)		0.90(.72–1.12)
4th and 5th		1.04(0.80–1.35)		1.05(0.80–1.36)
6th and above		0.97(.72–1.3)		0.96(0.71–1.31)
Religion				
Protestant (reference)			1	1
Muslim			1.33**(1.08–1.64)	1.45**(1.12–1.88)
Orthodox			0.98(.79–1.22)	0.94(0.72–1.23)
Others(catholic, traditional)			1.41*(1.01–1.97)	1.66*(1.0–2.57)
Place of residence				
Urban (reference)			1	1
Rural			1.49***(1.22–1.83)	1.29*(1.06–1.58)
Region				
Addis Ababa (reference)			1	1
Afar			1.84***(1.19–2.85)	1.00(0.58–1.72)
Amhara			3.11***(2.09–4.63)	1.88*(1.16–3.07)
Oromia			1.54*(1.04–2.29)	1.04(0.64–1.69)
Somali			0.87(0.57–1.34)	0.71(0.42–1.22)
Benishangul			2.11***(1.38–3.21)	1.75*(1.10–2.76)
SNNPR			2.01*(1.34–3.02)	1.35(0.82–2.22)
Gambella			1.13(0.73–1.77)	0.86(0.50–1.49)
Harari			1.39(0.89–2.15)	0.92(0.54–1.57)
Tigray			1.99**(1.29–3.01)	1.76*(1.01–2.92)
Dire Dawa			1.41*(1.01–1.97)	1.23(0.72–2.11)
Type of drinking water				
Unimproved (reference)			1	1
improved			1.01(0.90–1.13)	1.05(0.90–1.22)
Community-level women poverty				
Low (reference)			1	1
high			1.20*(1.03–1.39)	0.92(0.75–1.12)
Community-level women institutional delivery				
Low (reference)			1	1
high			0.80**(0.68–0.94)	0.89(0.72–1.09)
Random effect				
Community-level variance(SE)	0.37***(0.046)	0.21***(.049)	0.19***(.032)	0.17***(0.047)
ICC (%)	10.10%	6.00%	5.40%	5.00%
MOR	1.78	1.54	1.51	1.47
PCV	reference	44%	49.80%	53.6%
Model fit statistics				
AIC	11,420.09	6373.387	11,050.830	6234.555
BIC	11,434.27	6578.387	11,199.350	6551.946
Log-likelihood	5708.045	3155.693	5504.414	3069.278

Note: *significant at *P < 0.05; ** *P* < 0.01; *** *P* < 0.001; *AOR* Adjusted Odds Ratio, *CI* Confidence Interval, *AIC* Akaike information criterion, *BIC* Bayesian information criterion, Model 1-Empty (null) model; Model 2- Only individual-level explanatory variables included in the model; Model 3-Only community-level explanatory variables included in the model; Model 4-Combined model; *PCV* Proportional Change in Variance, *MOR* Median Odds Ratio

In contrast to the above, variables such as age of mother, age of mother at first birth, place of delivery, number of antenatal care (ANC) visits, father’s education level, mass media exposure and birth order of the last birth had no significance effect on childhood stunting (*P* ≤ 0.05) after adjusting for alternative individual- and community-level variables within the model (Table 1).

### Multivariable multilevel logistic regression analysis of community-level factors

During the multivariate multilevel logistic regression analysis, the community-level related factors such as religion, region, and place of residence were independently associated with log odds of childhood stunting among under-five children (*P* ≤ 0.05).

The likelihood of childhood stunting were 88% (AOR = 1.88, 95%CI: 1.16–3.07), 76% (AOR = 1.76, 95%CI: 1.01–2.92), and 75% (AOR = 1.75, 95%CI: 1.10–2.76) higher from the regions of Amhara, Tigray, and Benishangul respectively compared to those from Addis Ababa.

Relative to children from Protestant families, those from Catholic families were 41% (AOR = 1.41, 95%CI: 1.01–1.97) more likely to be stunted. By the same token, those from Muslim families were 33% (AOR = 1.33, 95%CI: 1.08–1.64) more likely to be stunted; and children from the rural communities were also 29% (AOR: 1.29, 95%CI: 1.06–1.58) more likely stunted compared to children from the urban communities, after controlling other variables of individual- and community-level within the model (Table 2).

### Results of the multilevel logistic regression model

In the empty model (Model-1), it had no individual- and community-level variables and it examined only the random and intercept variable. In the course of the analysis, there was significant variation in the log odds of childhood stunting across the communities (σ^2^_u0_ = 0.37, *P* < 0.001, 95%CI: 0.28–0.47). This variation remained significant after controlling the individual- and community-level factors in all models (Table 2). In Model-2 (individual model), it was also found significant variation in log odds of being stunted across the communities (σ^2^_u0_ = 0.21, *P* < 0.001, 95%CI: 0.13–0.33). According to the intra-community correlation coefficient implied, only 6% of the variance in the childhood stunting could be attributed to clustering effects (unexplained variation). In the log odds of being stunted across communities, 44% of the variance was explained by individual-level factors.

Model-3 (community model) examined the community-level factors of interest. There was significant difference in the log odds of being stunted across the communities (σ^2^_u0_ = 0.19, *P* < 0.001, 95%CI: 0.13–0.26); and the intra-community correlation coefficient implied by the estimated component variance was only 5.40% of the variance in childhood stunting that could be attributed to clustering effects. In the log odds of being stunted in the communities, 49.80% of the variance was explained by community-level factors.

Model-4 examined the individual- and community-level factors of interest. There was significant difference in the log odds of being stunted in the communities (σ^2^_u0_ = 0.17, *P* < 0.001, 95%CI: 0.10–0.29). In the log odds of being stunted variance across communities, 53.6% of the variance was explained by individual- and community-level factors combined.

### Model fit statistics

The AIC and BIC values of Model-1, Model-2, Model-3 and Model-4 were found to be 11,420.09, 6373.387, 11,050.830, 6234.555, and 11,434.27, 6578.387, 11,199.350, 6551.946 respectively (Table 2). Lower values indicate the goodness of fit of the multilevel model. The smallest values of Log-likelihood, AIC, and BIC were observed in model 4 and this implies that model-4 for childhood stunting was a better explanatory model. This also suggests that the addition of the community compositional factors increased the ability of the multilevel model in explaining the variation in childhood stunting between the communities.

### Multi-collinearity

Multi-collinearity amongst the individual- and community-level candidate explanatory variables was tested using the Variance Inflation Factor (VIF). In the current study, the mean VIF value was estimated to be 1.55 showing the absence of multi-collinearity in the models.

## Discussion

In this study, the prevalence of stunting was found to be 38.39% in Ethiopian under-five children. At the individual-level factors such as size of the child at birth, wealth index, education of the mother, birth type, BMI, sex and age of the child were found significant factors. Similarly, community-level factors such as religion, place of residence and region were found significant factors. The study indicated that the proportion change in variance of the full model was responsible for about 53.6% in the log odds of childhood stunting in the communities. The outcomes of median odds ratio, a measure of unexplained cluster heterogeneity, is 1.78, 1.54, 1.51, and 1.47 in models 1, 2, 3 and 4 respectively. Hence the results of median odds ratio revealed that there is unexplained variation between the clusters of the community**.** The ICCs results were also found to be above 2% of the total variance of childhood stunting in all models (Table 2). An ICC equal or greater than 2% is an indicative of significant group-level variance which is a minimum precondition for a multilevel study design [30].

In the present study, the childhood stunting was found to be significantly associated with the age of the child; as the child’s age increases the risk of being childhood stunted increases. Similar studies were reported in Bangladesh, Madagascar and Malawi [31–33]. It could be due to the inappropriate and late introduction of low nutritional quality supplementary food [34], and a large portion of guardians in rural areas are ignoring to meet their children’s optimal food requirements as the age of the child increases [35]. In addition, the lower odds of a breastfeeding rate of 0–11 month may indicate that exclusive and continuous breastfeeding has protective impacts for up to 1 year as defined by the WHO [36].

Small birth size children are usually born from low socioeconomic status and poor health [37, 38]. In the current study also confirmed that birth size and type of birth were found statistically significant. By the same token, study results confirmed that the probability of multiple births would be shrunk and low weight in similar to other studies [39, 40]. Multiple births involve birth defects like premature birth, birth weight, cerebral paralysis, all of which can inhibit child growth [41].

In the current study, male children were more likely to be stunted compared to their female groups of a comparable socioeconomic background similar to previous studies conducted in sub-Saharan Africa, Ethiopia and India [9–11, 39, 42, 43]. Gender difference in childhood stunting was more likely to be found in environments wherever there is stresses like continual infections and exposure to toxins and air pollutants [44]. On the contrary, another study from India showed that female children were more likely to suffer from childhood stunting than boys [45]. This might be due to the reason that breastfeeding duration was the lowest for daughters as their parents were trying for a son [46].

The childhood stunting was found to be inversely related to the mother’s level of education. This is in line with previous finding from developing countries [47]. These findings demonstrate the importance of the education of girls as alternative strategy to beat the burden of childhood stunting and to push sensible feeding practices for young children. Higher levels of maternal education can also reduce childhood stunting through other ways, such as increased knowledge of sanitation practices and healthy behaviors [48]. Children from rich mothers in wealth index were also positively associated with reducing childhood stunting. Studies conducted in Bolivia and Kenya found that less stunted children born to women with a high level of education and to women from high wealth households [49, 50].

In this study, low maternal BMI was found to be negatively associated with childhood stunting. This is also supported by the study conducted in Colombian school children [51] and in Southern Ethiopia [52]. A study from Brazil also suggested that maternal nutritional status was associated with child nutritional status [53]. According to Akombi et al. [24], the prenatal causes of child sub-optimal growth are closely related to maternal under nutrition, and are evident through low maternal BMI which predisposes the fetus to poor growth leading to intrauterine growth retardation; this in turn, is strongly associated with small birth size and low birth weight.

This study revealed that childhood stunting cannot be entirely explained by individual-level factors. The study suggested that children from Muslim, Catholic, and other traditional religion background were more likely to be stunted compared to children from communities with protestant families in line with previous studies in Ghana, Ethiopia, and India [54–56]. This may be due to some cultural factors, which are represented by the major religions in these countries [57].

The present findings suggested that children from Amhara, Benishangul, and Tigray communities were more stunted compared to children from Addis Ababa which is similar to previous studies that compared regional variations in nutritional status [58, 59]. This difference may be from the differential nutritional intake, the difference in the environment, socio-economic, and cultural differences [19].

Our study showed that children whose parents reside in rural areas had higher odds of childhood stunting than the urban areas. Possible explanations may be due to better-equipped urban health-care systems and higher access to health-care facilities. Urban populations usually have a higher educational level, economic status, and employment opportunities. [60]. This finding is similar with results from a cross-sectional study carried out in Mozambique, and Iran [61, 62]. On the other hand, in several countries, the rate of stunting in children living in slums is higher than in the rest of urban areas or rural areas [63].

### Limitations

Since the data was cross-sectional type, it could not show causal inferences in relation to individual- and community-level factors with childhood stunting. Another limitation is the use of secondary data which has restricted ability to include other variables such as healthcare indicators and dietary aspects in relation to childhood stunting. Management of missing data was also ignored.

## Conclusions

This study showed that both individual- and community-level factors determined childhood stunting among under-five children in Ethiopia. At the individual level, an increased age of the child, gender parity (being male), smaller size of the child at birth, lower BMI of the mother, poor wealth quintiles of the household, children of mothers with no education, and multiple births were found significant in determining childhood stunting. At community-level, children from communities of Amhara, Tigray, and Benishangul suffer more from childhood stunting as compared to Addis Ababa’s children., Children from Muslim, Catholic and other traditional families had higher log odds of stunting relative to children from the Protestant families. Moreover, children residing in rural Ethiopia were more likely to be stunted relative to urban dwellers. Thus, public health interventions targeting childhood stunting should focus on both the individual and community-level factors linked to maternal nutrition at large and child nutritional status.

## Data Availability

Data are available from the 2016 Ethiopian Demographic Health Survey Institutional Data Access/ Ethics Committee for researchers who meet the criteria for access to confidential data. Now it is available and can be obtained from the corresponding author (Mekonnen Haileselassie, Email: mekonnen210@yahoo.com).

## Abbreviations

AIC: Akaike information criterion
ANC: Antenatal Care
AOR: Adjusted odds ratio
BIC: Bayesian information criterion
BMC: Body Mass Index
CI: Confidence interval
ECSA: Ethiopian Central Statistical Agency
EDHS: Ethiopian Demographic and Health Survey
ICC: intra-cluster correlation coefficient
MOR: Median Odds Ratio
PCV: Proportional Change in Variance
WHO: World Health Organization
